# The Pak4 protein kinase is required for oncogenic transformation of MDA-MB-231 breast cancer cells

**DOI:** 10.1038/oncsis.2013.13

**Published:** 2013-06-03

**Authors:** L E Wong, N Chen, V Karantza, A Minden

**Affiliations:** 1 Susan Lehman Cullman Laboratory for Cancer Research, Department of Chemical Biology, Ernest Mario School of Pharmacy, Rutgers, State University of New Jersey, Piscataway, NJ, USA; 2 Cancer Institute of New Jersey, New Brunswick, NJ, USA; 3 Division of Medical Oncology, Department of Internal Medicine, University of Medicine and Dentistry of New Jersey, Robert Wood Johnson Medical School, Piscataway, NJ, USA; 4 Department of Chemical Biology, Rutgers, State University of New Jersey, Piscataway, NJ, USA

**Keywords:** breast cancer, Pak4, acini, tumorigenesis

## Abstract

The Pak4 protein kinase, normally expressed at low level in the mammary gland, is commonly overexpressed in breast cancer. Overexpression of Pak4 transforms mouse mammary epithelial cells *in vitro* and renders these cells tumorigenic in athymic mice *in vivo*. Here we show that Pak4 is also required for oncogenic transformation of the human breast cancer cell line MDA-MB-231. These high Pak4-expressing human breast cancer cells form highly disorganized three-dimensional (3D) structures *in vitro* and readily give rise to orthotopic xenograft tumors in nude mice. We have found that when Pak4 levels are reduced, MDA-MB-231 cells exhibit decreased proliferation and migration *in vitro*, as well as gross restoration of normal 3D mammary acinar organization, the latter in association with a strong induction of apoptosis. Similarly, Pak4 knockdown suppresses MDA-MB-231 breast xenograft tumor formation in nude mice *in vivo*. These results indicate that Pak4 has a key role in the oncogenic transformation of breast cells.

## Introduction

The Pak family of serine/threonine kinases are important signaling proteins implicated in many cellular functions including cell proliferation, migration and cytoskeletal organization.^1^ The Pak family consists of six members. These include group A; Paks 1, 2 and 3, and group B; Paks 4, 5 and 6.^1^ The Paks have an amino terminal GTPase-binding domain, which can bind to the Rho GTPases Cdc42 and Rac, and a carboxyl terminal serine/threonine kinase domain. The group A and B Paks have approximately 50% amino-acid identity in the GTPase-binding domain and kinase domains, but differ throughout their other domains.^1^ Pak4 has important roles in cell proliferation, survival, cell shape and animal development. Pak4 has been implicated in several types of cancer,^2, 3, 4, 5^ and strong links have been observed between Pak4 and breast cancer.^4, 5, 6, 7, 8^ Pak4 is overexpressed in breast cancer cell lines^4, 5, 9^ and in primary human breast tumor and rat mammary tumor samples,^4^ but it is barely detectable in normal tissue.^4^ In the MCF10A cell progression series, which consists of mammary epithelial cells that range from nontransformed to highly tumorigenic, Pak4 levels are highest in the more tumorigenic cells.^8^ The chromosomal region containing Pak4, 19q13.2, is frequently amplified in aggressive breast cancers with basal-like features.^6^

In addition to being overexpressed in breast cancer, Pak4 may also be a driving force in the disease, and this has been demonstrated recently using an immortalized mouse mammary epithelial cell (iMMEC) system.^5^ Mammary glands consist of ordered structures containing spherical acini, and tumorigenesis is associated with disruption of these structures.^10, 11^ iMMECs, which were isolated from normal mouse mammary glands, serve as a good *in vitro* model for studying mammary tissue architecture.^12^ These cells can be grown under conditions where they form three-dimensional (3D) structures, by growing them on a layer of basement membrane.^12^ When grown in 3D culture, iMMECs form spherical acini, mimicking those seen in normal breast epithelia.^11^ The formation of these acini follows a temporal sequence of events, in which a polarized outer layer of cells forms, and an inner, non-polarized group of cells dies by apoptosis, resulting in the formation of a hollow lumen.^11^ The resulting structures recapitulate normal mammary tissue architecture. iMMECs generated from wild-type mice have nearly undetectable Pak4 levels, consistent with the negligible Pak4 levels seen in normal mammary gland. When iMMECs are stably transfected with wild-type Pak4, the resulting acinar structures exhibit increased cell proliferation and survival, filling of the luminal space with cells, increased acinar size, loss of cell polarity and a larger outer layer of epithelial cells.^5^ These changes are all hallmarks of precancerous conditions and early tumor stages, such as atypical hyperplasias and ductal carcinoma *in situ* (DCIS). Even more importantly, the Pak4-expressing iMMECs form tumors when implanted into the mammary fat pads of nude mice,^5^ providing strong evidence that Pak4 overexpression drives mammary tumorigenesis. The oncogenes Her2/Neu and mutant H-Ras also cause iMMECs to become tumorigenic^13^ and, interestingly, they lead to elevated Pak4 levels.^5^ Pak4 thus appears to be a key downstream target by which oncogenes promote mammary tumorigenesis. In addition to Pak4, other Pak family members are also overexpressed in breast cancer, particularly Pak1.^14, 15, 16, 17, 18, 19, 20^ Pak4 appears to be unique, however, in that wild-type Pak4 alone is sufficient to cause tumorigenesis in mice when overexpressed.^4, 5^

The expression pattern of Pak4 and the finding that its overexpression leads to transformation of mammary epithelial cells *in vitro* and mammary tumorigenesis *in vivo* make Pak4 a promising therapeutic target. However, for Pak4 to be an effective target, it is important to demonstrate that Pak4 is not only sufficient but is also required in mammary tumorigenesis. The MDA-MB-231 breast cancer cells, like many human breast cancer cell lines, have high Pak4 levels.^9^ In this study, we used siRNA to reduce Pak4 levels in MDA-MB-231 cells and found that reduction of Pak4 expression led to dramatic changes in cell growth and morphology that were consistent with at least partially reversing the malignant transformation process. These results indicate that Pak4 has a key role in the oncogenic transformation of breast cells, and support the idea that Pak4 or Pak4-mediated pathways may become important drug targets for breast cancer treatment.

## Results

### Pak4 knockdown in MDA-MB-231 breast cancer cells

**Figure 1:**
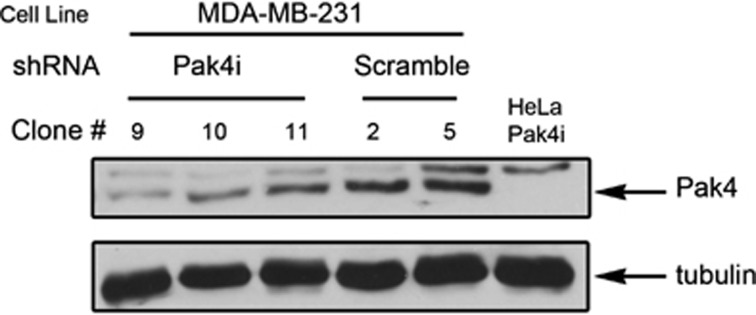
Analysis of Pak4 protein levels in MDA-MB-231 cells stably expressing Pak4 shRNA (Pak4i) or control shRNA (Pak4 scramble). Reduction of Pak4 expression in puromycin-resistant clones was assessed by western blot analysis. Cells were harvested in lysis buffer, and equal amounts of total cell protein were separated by SDS–PAGE and immunoblotted using the indicated antibodies. The results were quantitated by densitometry analysis, and the amount of Pak4 was normalized to a tubulin-loading control. Lanes 1–3: Pak4 shRNA expressing clone numbers 9, 10 and 11. Lanes 4 and 5: control shRNA expressing clones 2 and 5. Lane 6: HeLa Pak4 shRNA cells, used as a control for the Pak4 shRNA. The top band is a nonspecific band and the bottom band is Pak4.

To stably reduce expression of Pak4 in MDA-MB-231 human breast cancer cells, the cells were infected with a retroviral vector containing a Pak4 short hairpin RNA (shRNA) sequence and a puromycin resistance marker, or with a control vector containing a random RNA sequence, as described in the Materials and methods section. Puromycin-resistant colonies were assessed by western blot analysis. The results were quantified by densitometry analysis, and the amount of Pak4 was normalized to a tubulin-loading control. The three knockdown clones showing the lowest levels of Pak4 and the two control clones showing the highest levels of Pak4 are shown (Figure 1). Clone 9, shown in lane 1, contained the lowest amount of Pak4 expression, approximately 40% of control levels. This clone, referred to as ‘Pak4i', was used in subsequent experiments.

### Pak4 knockdown reduced proliferation of MDA-MB-231 cells

**Figure 2:**
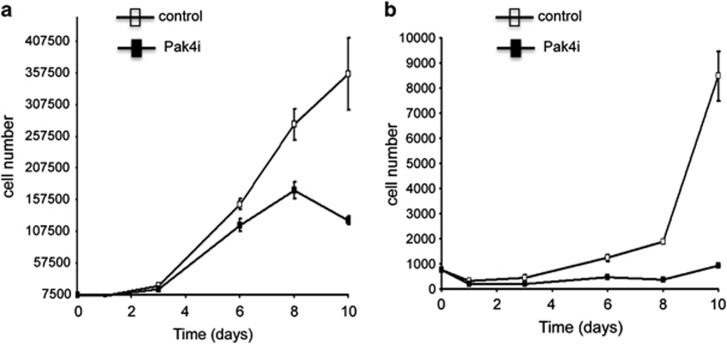
Pak4 knockdown reduces proliferation of MDA-MB-231 cells and increases serum dependence. The effect of reduced Pak4 expression on cell proliferation was assessed by growth curve assay. Pak4i and control cells were plated at a density of 7500 cells/well in 12-well cluster plates. Triplicate samples of each cell line were counted at each time point, with viable cell counts determined by trypan blue exclusion. The cell numbers of the triplicate samples were averaged and plotted. Error bars represent s.e.m. (**a**) Growth in 10% serum. (**b**) Growth in 0.5% serum.

The effect of the reduction of Pak4 expression on proliferation of MDA-MB-231 cells was assessed by growth curve assay (Figure 2). Pak4i and control cells were plated at low density in tissue-culture cluster plates in growth medium containing 10 or 0.5% fetal bovine serum. Beginning at day 1 post seeding, cell number was determined by counting viable cells at intervals of 2–3 days until day 10. In medium containing 10% serum, Pak4i cells grew significantly slower than control cells. In low serum conditions, however, the effect was even more striking. Under these conditions, the Pak4i cells essentially underwent growth arrest over the course of the assay. During this time, the control cells grew steadily with a remarkable increase in growth rate between days 8 and 10. Reduced expression of Pak4 in MDA-MB-231 cells is therefore accompanied by a reduction in the rate of cell growth and increased serum dependence.

### Pak4 knockdown impairs motility of MDA-MB-231 cells

**Figure 3:**
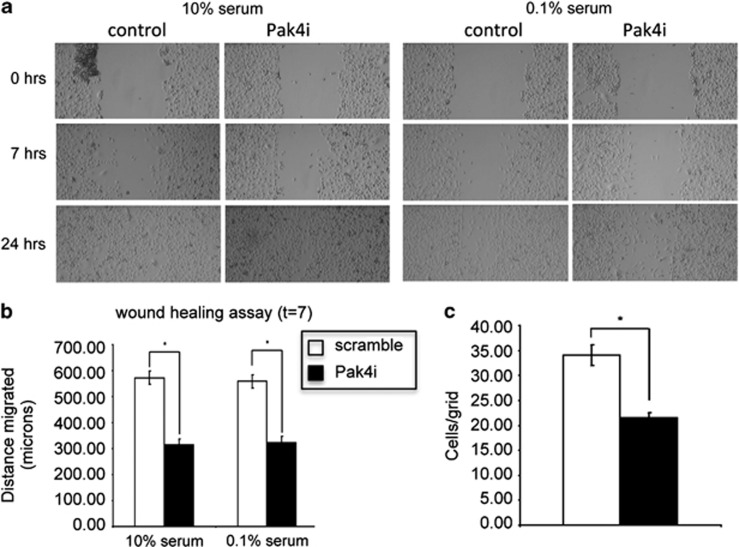
Pak4 knockdown impairs motility of MDA-MB-231 cells. The effect of reduced Pak4 expression on cell motility was assessed by wound-healing assay. Confluent monolayers of Pak4i (knockdown) and control cells grown in six-well cluster plates were scratched using sterile pipet tips, then allowed to recover in medium containing either 10 or 0.1% fetal bovine serum. Phase contrast micrograph images were recorded at the time of wounding (0 h), and at 7 and 24 h (**a**). (**b**) Distance migrated at 7 h was determined by measuring the width of the wounded area and subtracting that from the original width of the wound. (**c**) Migration at 24 h in 0.1% serum was determined by counting the number of cells that had migrated into the wounded area. Ten representative grids in each wounded area were counted and the results averaged and plotted. Error bars represent s.e.m. Significance values were calculated using a one-tailed unpaired *t*-test, with *P*<0.05 considered significant (represented by *).

MDA-MB-231 cells are derived from metastatic breast cancer and are highly invasive. Because Pak4 has been implicated in cytoskeletal organization,^21^ which is an important process during cell migration, we tested whether Pak4 has an essential role in MDA-MB-231 cell motility (Figure 3). Wound-healing assays were performed under normal serum and serum starvation conditions to assess cell migration. By 7 h post wounding, significant differences could be seen between Pak4i and control cells. Quantitation of the results shown in Figure 3a shows that after 7 h, Pak4i cells have migrated an average of approximately 320 , whereas the control cells migrated approximately 75% farther. There was no significant difference in migration in normal vs serum starvation conditions, suggesting that the difference between Pak4i and control cells was not due to differences in cell proliferation (Figure 3b).

### Knockdown of Pak4 restores acinar formation in MDA-MB-231 cells grown in 3D culture

**Figure 4:**
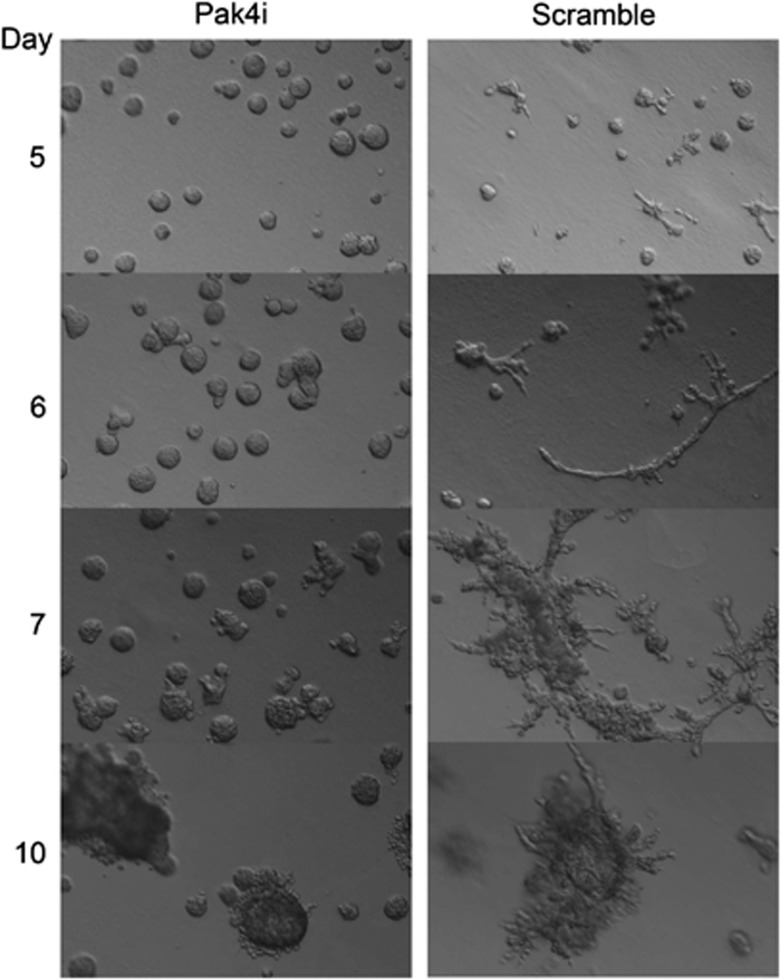
Pak4 knockdown restores ordered structures of acini formed by MDA-MB-231 cells grown in 3D culture. Pak4i and control (scramble) cells were grown in on a layer of basement membrane in medium containing 2% Matrigel. Phase contrast micrograph images were taken at the indicated time points.

Compared with traditional two-dimensional cell culture, 3D basement membrane cell culture is a more physiologically relevant system for studying glandular structures. When grown in 3D cultures, noncancerous mammary epithelial cells such as iMMECs or MCF10A cells form organized spherical acini. Pak4 overexpression disrupts acinar morphogenesis, and, similarly, human breast cancer cells form highly disorganized structures when grown in 3D culture. This is consistent with the disruption of normal tissue architecture seen in cancer. To determine whether reduction of endogenous Pak4 expression would restore a normal program of acinar development in MDA-MB-231 cells, Pak4i and control cells were grown on a layer of basement membrane in a 3D culture system as previously described^12^ (Figure 4). The cultures were monitored by phase contrast microscopy. By day 5, the structures formed by the control MDA-MB-231 cells began to lose their globular shape, forming outgrowths that appear to join the structures together, eventually forming massive interconnected structures typical of cancer cells. In contrast, although the Pak4i structures continued to grow, they never completely lost their globular shape.

**Figure 5:**
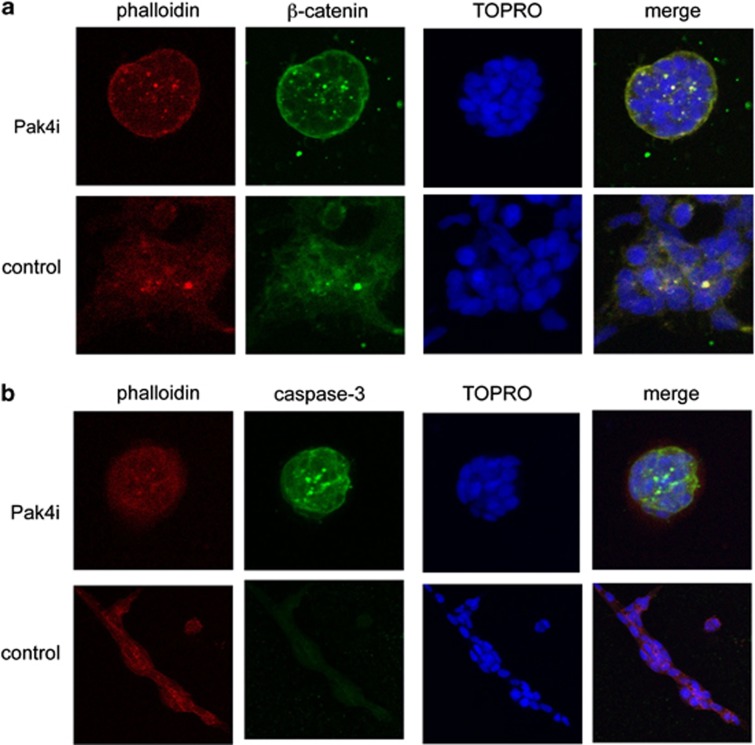
Knockdown of Pak4 restores apoptotic activity and acinar lumen formation in MDA-MB-231 cells grown in 3D culture. Acinar structures were fixed and processed for immunofluorescence, and stained with anti-β-catenin to visualize epithelial cell–cell junctions (**a**), or anti-cleaved caspase-3 primary antibodies to visualize apoptosis (**b**) and fluorescein isothiocyanate-conjugated secondary antibodies (green). Acini were counterstained with Alexafluor-conjugated phalloidin (red) to visualize the actin cytoskeleton and TOPRO (blue) to visualize cell nuclei.

Pak4i and control acinar structures were subjected to immunofluorescence and examined by confocal microscopy (Figures 5a and b). At day 6, the control cells exhibited the expected disorganized structure. β-catenin, which is normally localized at cell–cell junctions, showed diffuse cytoplasmic staining, and apoptotic cells, as assessed by staining with cleaved caspase-3 antibody, could not be seen. In contrast, the Pak4i cells formed structures that appeared to develop into organized acini. β-catenin was localized to the cell membrane, and most strikingly, apoptotic cells could be seen in the centers of these structures. These results indicate that reduction of Pak4 drastically restores normal acinar structure, accompanied by increased apoptosis of the acinar luminal cells.

### Pak4 knockdown reduces tumorigenesis in mice

**Figure 6:**
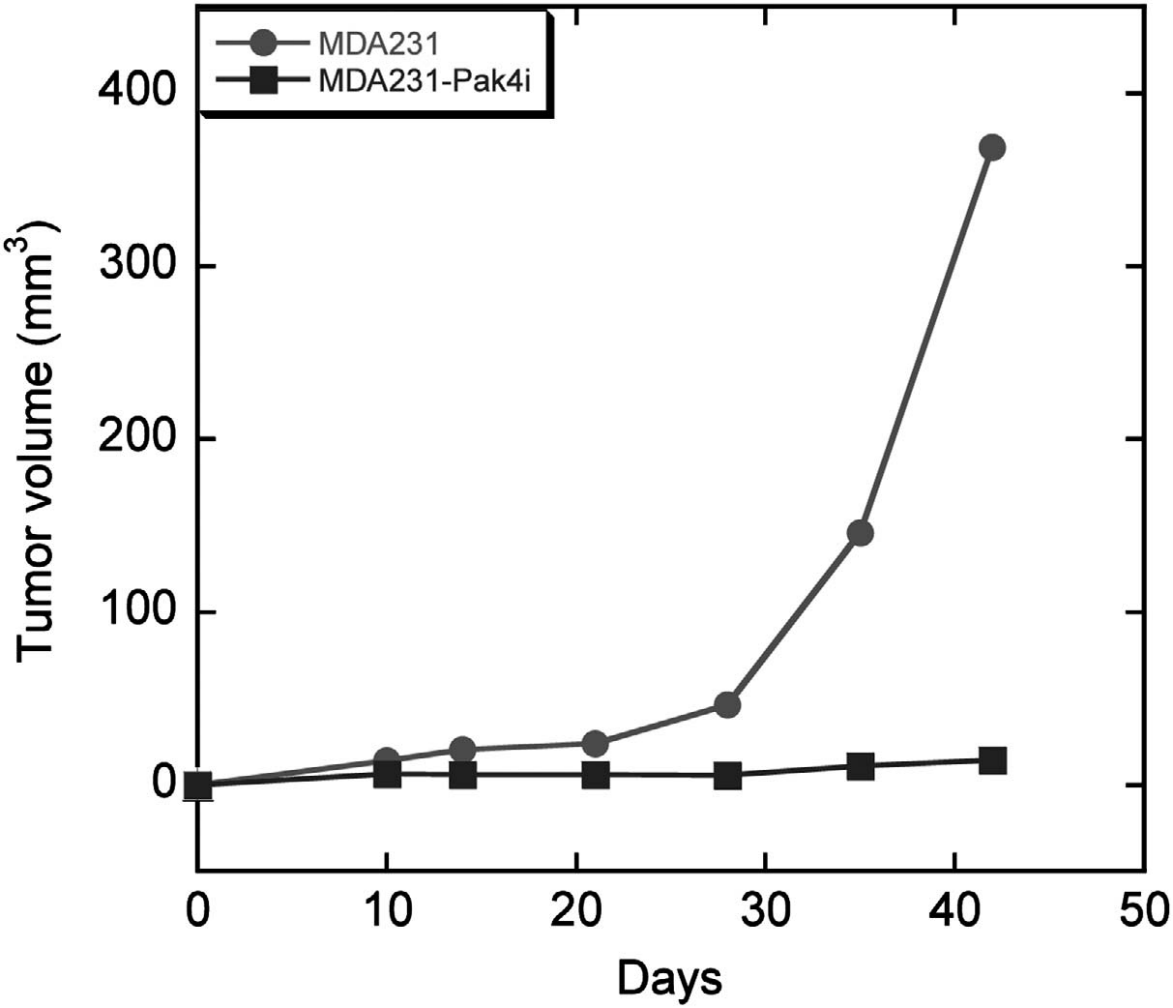
Pak4 knockdown impairs the ability of MDA-MB-231 cells to form tumors. Pak4i and control cells were implanted into the mammary fat pads of athymic mice. Tumors were observed and measured up to 40 days post implantation.

To test whether reduction of Pak4 levels blocked tumorigenesis in mice, the MDA-MB-231 control and knockdown cells were implanted into the mammary fat pads of athymic mice. Tumors were then observed and measured up to 40 days post implantation. Strikingly, although the control cells formed large tumors, knockdown of Pak4 almost completely blocked the formation of tumors (see Figure 6).

## Discussion

The Pak4 protein kinase was previously shown to be sufficient to lead to transformation of mammary epithelial cells. The goal of this study was to determine whether Pak4 is also necessary for mammary cell tumorigenesis, as this would make it an attractive candidate for a drug target. Cancer is a highly complex disease, and while not one signaling pathway is likely to be the one and only mediator of any type of cancer, our results indicate that blocking Pak4 significantly reduced tumorigenesis in our *in vitro* model and in mice, as well as cell migration, which can be closely linked with metastasis.

Knockdown of Pak4 dramatically reduced proliferation and migration of breast cancer cells, but the most dramatic finding was that Pak4 knockdown largely restored normal acinar structure. When MDA-MB-231 cells are grown under 3D conditions, they form highly disorganized structures, typical of breast cancer cells. After 5 days in culture, acinar structures formed by the control cells began to lose their organized globular shape, but when Pak4 levels were reduced, the organized globular structures of the acini were largely restored. An especially important result was seen when acini were stained with cleaved caspase-3 (as a marker of apoptosis). Typically, as acini develop the cells in the center undergo apoptosis, resulting in the formation of a hollow lumen. Loss of apoptosis is common in cancer, and in acini this can lead to filling of the luminal space. Strikingly, although apoptosis appeared to be minimal in the MDA-MB-231 cancer cells, apoptosis was markedly restored when Pak4 was knocked down. Pak4 has previously been shown to be associated with increased cell survival and decreased apoptosis when it is overexpressed.^22, 23, 24^ Increased survival, combined with increased cell migration could help explain why Pak4 overexpression is associated with cancer.

We have found that Pak4 has an important role in mammary cell transformation, but other Pak family members have also been implicated in breast cancer. Group A Paks are also involved in breast cancer and Her2Neu signaling, particularly Pak1.^14, 15, 16, 17, 18, 19, 20^ The focus of study is Pak4, but in the future it will be important to use similar techniques to examine the roles for other Pak family members in breast cancer, and to determine whether Pak4 may act in conjunction with other Paks in cancer, or if different Pak family members operate in different types of breast cancer or at different stages. This will be important for the future development of the right kinds of drugs or drug combinations that target specific types of breast cancer.

## Materials and methods

### Reagents and antibodies

Rabbit polyclonal Pak4 and cleaved caspase-3 antibodies were from Cell Signaling Technologies (Danvers, MA, USA). Mouse monoclonal tubulin antibody and horseradish peroxidase-conjugated goat anti-mouse and anti-rabbit antibodies were from Sigma Life Sciences (St Louis, MO, USA). Cy3-coupled anti-mouse and fluorescein isothiocyanate-coupled anti-rabbit antibodies were from Jackson Immunoresearch Laboratories (West Grove, PA, USA). Alexafluor-coupled phalloidin and TOPRO were from Molecular Probes (Grand Island, NY, USA). Reduced growth factor Matrigel was purchased from BD Biosciences (Franklin Lakes, NJ, USA).

### Cell culture and transfection

MDA-MB-231 cells (gift from Dr Nanjoo Suh) were cultured in DMEM/F12 (Cellgro). Amphitropic Phoenix retroviral packaging cells were grown in Dulbecco's modified Eagle's medium. All media were supplemented with 10% fetal bovine serum, 50 U/ml penicillin and 50 μg/ml streptomycin. Puromycin (1.5 μg/ml) was added for selection and maintenance of stable cell lines. 3D cultures of MDA-MB-231 cells were carried out as previously described.^12^ Mammary acinar structures were grown in DMEM/F12 medium containing 2% Matrigel. All cell lines were grown at 37 °C in 5% CO_2_.

### Construction of stable Pak4-knockdown cell lines

The 64-mer DNA oligonucleotide encoding the siRNA sequence targeting a linker region between the regulatory domain and the kinase domain in the Pak4 cDNA has been previously described.^24^ These nucleotides, as well as scrambled control nucleotides, were synthesized by IDT-DNA. pSuperRetro.puro-Pak4 and pSuperRetro.puro-Scramble were constructed by ligation of the annealed nucleotides into the *Bgl*II and *Hin*dIII sites of the pSuperRetro.puro vector (gift from Dr Albert B. Reynolds). These vectors were used to transfect amphitrophic Phoenix packaging cells according to the Nolan lab protocols. Retroviral supernatants were collected and used to infect MDA-MB-231 cells according to Nolan lab protocols. The cells were selected with puromycin and 20 colonies picked from each transduction. The expression of Pak4 was assessed by western blot. The results were quantified by densitometry analysis of the blots, and the amount of Pak4 was normalized to the tubulin-loading control. The three knockdown clones showing the lowest Pak4 expression and the two control clones showing the highest levels of Pak4 are shown (Figure 1). Clone 9, shown in lane 1, has the lowest amount of Pak4 expression, approximately 40% of control levels. This clone, referred to as ‘Pak4i', was used in subsequent experiments.

### Cell proliferation assay

The effect of Pak4 knockdown on cell proliferation under normal and low serum conditions was assessed by performing growth curves. Cells were plated at a density of 7500 cells/well in 12-well cluster plates (Falcon, BD Bioscience). Growth medium was renewed every 3 days during the course of the assay. Triplicate samples of each cell line were counted at each time point. The cells were trypsinized, centrifuged at 500 *g* and resuspended in trypan blue. Viable cells were counted using a hemocytometer. The cell numbers of the triplicate samples were averaged and plotted using Microsoft Excel (Microsoft). Error bars represent s.e.m.

### 3D morphogenesis and indirect immunofluorescence

Before fixation and staining, 3D cultures were examined by phase contrast microscopy using an Olympus CK2 microscope, with images recorded after 5, 6, 7 and 10 days. Mammary acinar structures were fixed and processed for immunofluorescence as previously described,^12^ at day 6. The fixed cultures were incubated with primary antibodies overnight at room temperature, washed and then incubated with Cy3- or fluorescein isothiocyanate-coupled secondary antibodies as well as Alexafluor-labeled phalloidin for 1 h at room temperature. Finally, the structures were stained with TOPRO, washed and mounted with Prolong Gold Anti-Fade (Molecular Probes). Fluorescent stained 3D cultures were examined using a Nikon Eclipse Ti confocal microscope (Nikon, Melville, NY, USA) using EZ-C1 viewer imaging software (Nikon).

### Western blots

Western blots and quantitation of western blot data were carried out as previously described.^25^ Primary antibodies were diluted into TBS/T containing 5% bovine serum albumin as follows: anti-Pak4, 1:1000 and anti-tubulin, 1:10 000.

### Wound-healing assay

MDA-MB-231 cells were plated in 12-well cluster plates at a density of 150 000 cells per well and allowed to grow to confluence. The medium was aspirated and the monolayers were wounded by scratching with a sterile pipette tip in a direction perpendicular to an orientation mark on the plate. After washing three times with serum-free medium, growth medium containing either 10 or 0.1% fetal bovine serum was added. Phase contrast microscopic images were recorded at the time of wounding (0 h), and at 7 and 24 h. The width of the open areas was measured using Photoshop (Adobe), and the results averaged. Graphical presentation of the results and statistical analysis were carried out using Microsoft Excel. Error bars represent s.e.m., and significance values were calculated using a one-tailed unpaired *t*-test, with *P*<0.05 considered significant.
